# Evaluating the Heterogeneity Effect of Fertilizer Use Intensity on Agricultural Eco-Efficiency in China: Evidence from a Panel Quantile Regression Model

**DOI:** 10.3390/ijerph19116612

**Published:** 2022-05-28

**Authors:** Mengyang Hou, Zenglei Xi, Suyan Zhao

**Affiliations:** 1 School of Economics, Hebei University, Baoding 071000, China; houmengyang@hbu.edu.cn (M.H.); hbuxzl@hbu.edu.cn (Z.X.); 2 Center of Resources Utilization and Environmental Conservation, Hebei University, Baoding 071000, China; 3 School of Management, Hebei GEO University, Shijiazhuang 050031, China; 4 Natural Resource Asset Capital Research Center, Hebei GEO University, Shijiazhuang 050031, China

**Keywords:** agricultural eco-efficiency (AEE), fertilizer use intensity (FUI), quantile regression model (QRM), heterogeneity, spatial lag

## Abstract

Chemical fertilizer is one of the most important input factors in agricultural production, but the excessive use of fertilizer inevitably leads to the loss of agricultural eco-efficiency (AEE). Therefore, it is necessary to explore the impact of fertilizer use intensity (FUI) on AEE. However, ordinary panel regression, based on the assumption of parameter homogeneity may yield biased estimation conclusions. In this regard, a panel quantile regression model (QRM) was constructed with the provincial panel data of China from 1978–2020 to test the difference and variation of this impact under heterogeneous conditions. The model was then combined with the spatial econometric model to explore the effect of the spatial lag factor. The results are as follows: (1) The QSM has unveiled a great improvement space for AEE that remains low overall, despite displaying a rising trend; the highest AEE is in the eastern region. (2) The FUI has a significant negative effect on AEE with the rise in quantiles, this negative effect tended towards weakening overall, although it rebounded slightly; it was stronger in areas with low AEE. It is necessary to consider the heterogeneous conditions in comparison with the average treatment effect of ordinary panel econometric regressions. (3) The impact of FUI shows significant variability in different economic sub-divisions and different sub-periods. (4) After considering the spatial effect of fertilizer use, the negative influence on local AEE had a faster decay rate as the quantile rose, but could produce a positive spatial spillover effect on AEE in neighboring areas. Local governments should dynamically adjust and optimize their fertilizer reduction and efficiency improvement policies according to the level and development stage of their AEE to establish a complete regional linked agroecological cooperation mechanism.

## 1. Introduction

Sustainable development of agriculture needs to have a coordinated relationship between inputs, resource consumption, and environmental protection. Chemical fertilizer, one of the most critical inputs in agriculture, has contributed greatly to grain production, especially in China, where the contribution rate of chemical fertilizer has reached 56.81% [1,2]. However, data released by the Ministry of Agriculture revealed that the fertilizer use intensity (FUI) in China in 2018 was around 0.067 kg per hm^2^, much higher than the world average of 8 kg per mu, despite a decreasing trend, and nitrogen, phosphate, and potash fertilizer are overapplied to varying degrees [3,4], with the utilization rate of these fertilizers at 33%, 24%, and 42%, respectively. Therefore, excessive and inefficient fertilizer use represents the price paid for the development of agriculture and calls for improved agricultural eco-efficiency (AEE), as this industry encounters increasingly limited growth space pumped by fertilizer [5]. The Central Document No.1 of the Communist Party of China (CPC) in 2021 also pinpointed the need to reduce the use of chemical fertilizer and increase the efficiency of its use in order to accelerate the green transformation of agricultural production. Still, the current widespread distortion of the chemical fertilizer factor market exerts a significant stimulating effect on the emission of fertilizer non-point source pollution [6], with the overuse of fertilizer dwindling the marginal contribution to grain production and exacerbating agricultural non-point source pollution emission [7,8,9]. The fallout for this is environmental harms such as water pollution [10], biodiversity loss [11] and soil quality decline [12], then decreased AEE, and the impeded attainment of the “double carbon goal” and green development in agriculture across the board [13,14,15].

It is of great practical significance to discuss the impact of fertilizer use on AEE, to rationally control the fertilizer use intensity (FUI) and promote carbon reduction and the green transformation of agriculture in the production process. In addition, from international experience, developed countries such as those in the EU or North America have shown a tendency to maintain a steady decrease after reaching the peak, achieving a balance between fertilizer use, agricultural efficiency, and the ecological environment. Therefore, studying the impact of fertilizer reduction behavior on agriculture in China is expected to provide a reference for other countries in terms of fertilizer use, agricultural production, and ecological sustainability.

Eco-efficiency refers to the coordinated relationship between inputs and outputs that meet the needs of human production, resources, and the environment within the carrying capacity of the earth, achieving a balance between economic benefit and environmental protection. It was first proposed by Schaltegger and Sturn [16] from Germany and promoted by the World Business Council for Sustainable Development (WBCSD) [17] and the OECD [18]. For food production, agricultural eco-efficiency (AEE) incorporates resource and environmental constraints into the analytical framework measuring agricultural production efficiency. This is to obtain as much agricultural output as possible with as little resource consumption and environmental pollution as possible under a certain combination of agricultural input factors to ensure the quality of farm products [19,20]. This requires attention to both the economic benefits of agricultural production activities and their resource and environmental constraints in order to achieve the dual goals of agricultural output growth and environmental management.

Current research revealed that chemical fertilizer fails to draw much attention to its ecological impact, though its contribution has been recognized as one of the largest among all input factors to the agricultural process [1,2]. Therefore, it is necessary to pay attention to the impact of chemical fertilizer use on AEE. In addition, due to the distinctions in the agricultural economic basis, input structure, resource availability, and geographical location, the varying characteristics of chemical fertilizer use affecting AEE under different conditions are also noteworthy. Given the above, this paper will explore these issues through macro panel data and econometric test models based on a review of the literature.

## 2. Theoretical Basis and Literature Review

With the reform of the urban–rural dual structure system and the gradual elimination of mobility barriers, rural laborers gradually move to urban areas and engage in non-farm activities [21]. This not only prompts the re-allocation of agricultural labor in farm households but also causes changes in the intensity and structure of agricultural production inputs, which, in turn, affects the agricultural productivity and ecological environment of farmland. As rational economic agents, farmers will weigh their profits against changes to the external environment to efficiently allocate production resources and maximize output. To avoid output reduction and efficiency loss due to rural labor mobility, in certain areas of cropland, farmers increase the use of fertilizer and other chemicals to ensure their agricultural output [22]. According to the theory of externality, fertilizer is a material factor with a negative externality. In the process of agricultural production, although increasing fertilizer input per unit area can improve the productivity of cropland and the output of agricultural products, excessive input and inefficient use also lead to increases in agricultural non-point pollution emission and agricultural carbon emission, resulting in negative externalities for farmland in terms of the ecological environment and the loss of AEE. In addition, fluctuations in fertilizer use and the flow of rural labor put agricultural production at risk of distortion and a mismatch in resources and allocation [23]. Increasing the input per unit area of material factors such as fertilizer can help increase agricultural productivity, but the mismatch of production factors may not only lead to a reduction in the scale of output but also a decrease in AEE. Because of this, fluctuations in fertilizer input per unit area would affect AEE, and the reduction of fertilizer use and the weakening of its input intensity, as well as the optimization of the factors structure, are the keys to ensuring agricultural production and improving AEE.

We also need to note that there are differences in endowment conditions, agricultural production bases, and factor input structures in different regions. There are certain differences in the AEE in regions with different fertilizer use intensities (FUIs), which would result in different effects of FUI for regions with different AEEs. From a development perspective, when the AEE is low, micro-farmers tend to give up some environmental benefits and improve agricultural output by increasing fertilizer input and other material factors per unit area. With the optimization and adjustment of factor structure and the improvement in AEE, the factor allocation structure tends to stabilize and the negative externalities of fertilizer inputs gradually come to the fore, farmers are more inclined to reasonably control the production factor inputs based on the protection of farmland environment. It can be seen that FUI has different effects at different development stages of efficiency level.

Current research on the relationship between fertilizer use and agricultural production has focused more on the beneficial effect of fertilizer input on grain yield. It has been confirmed that fertilizer use has a significant contribution to an increase grain yield, with a contribution rate of more than 40% [1,24]. Fertilizer overuse has heightened grain yield [10,25], but the diminishing marginal effect has led to a continuous decrease in the actual grain yield growth rise caused by fertilizer [5,15]. With regard to the degree of fertilizer overuse, Wu et al. found that not all wheat growers in North China were concerned by excessive use of chemical fertilizer after analyzing the optimal fertilizer use amount and its deviation as well as the influencing factors for small-scale and large-scale farmers [26]. Wei and Li reported fertilizer overuse in almost all provinces over the study period when evaluating the fertilizer overuse index regarding arable agricultural production in China and examining regional variations in fertilizer overuse [27]. Qiu et al. measured the extent of fertilizer overuse in China based on survey data, and pointed out that the risk aversion of a farmer the essential reason for fertilizer overuse [28]. Shi and Zhu (2016) observed that the current amount of fertilizer use in China has exceeded the optimal use rate in the economic sense, and that farmers overapplied fertilizer both in wheat, maize, and rice production, with the average over-input of chemical fertilizer for the three grain crops reaching over 30%. Some scholars have also revealed the low efficiency of chemical fertilizer overuse [29]. Hu et al. estimated the fertilizer use efficiency based on the survey of farm households and found that the average FUE of whole samples was only 0.60, much lower than the average technical efficiency, indicating that on average, half of the fertilizer utilized in China was excessive [30]. Wu measured fertilizer use efficiency at the farmer level in China based on farmer survey data and found that it was low, with two-thirds of fertilizer input being excessive [31]. Bai et al. (2019) examined the fertilizer use efficiency of apple production by adopting panel data of eight major apple production provinces in China [32]. The FUE score ranged from 0.002 to 0.878, with a mean value of 0.472, and possessed greater variability than technical efficiency. In recognition of the problems caused by excessive fertilizer use, the Ministry of Agriculture and Rural Affairs of the PRC has formulated the “Action Plan for Zero Growth in Fertilizer Use by 2020” to promote fertilizer reduction and efficiency.

The negative effects to the ecological environment, such as severe agricultural non-point source pollution and soil acidification, caused by the excessive and inefficient use of chemical fertilizer, has gradually attracted much attention [33]. The study of Ge and Zhou (2012) shows that the widespread distortion of the chemical fertilizer market in China promotes the emission of agricultural non-point source pollution. After calculating the load of agricultural non-point source pollution in different irrigate amounts and comparing with fertilization schedules in paddy fields, Wang et al. unveiled a positive correlation between the fertilization and non-point pollution load, with the fertilizer pollution load seeing a record bump when turning green to tillering [34]. Some other literature has focused on the relationship between fertilizer use and agricultural economic growth. Li and Zhang found a typical inverted U-shaped curve relationship between fertilizer input, non-point source pollution, and economic growth, based on an environment Kuznets curve (EKC) test of inter-provincial panel data [35]. Zhang and Hu examined the environmental Kuznets curve between economic development and excess nitrogen pollution in Jiangsu Province with a significant inverted U-shaped curve relationship discovered [36]. At the same time, the results of the EKC test by Cao and Li (2011) showed no inverted U-shaped EKC relationship between them, with different study areas, samples, and agricultural pollution indicators leading to different EKC test results [37]. Zhang et al. investigated the relationship between rural non-point source pollution and economic development in the Three Gorges Reservoir area using the EKC hypothesis [38].

Research on the contribution of fertilizer to grain yield, the extent of fertilizer overuse, and the economic benefits of fertilizer input abound, with more scrutiny being afforded the latter aspect. However, fertilizer’s impact on the ecological efficiency of agriculture still requires further attention. The implication of similarity in diverse regions and levels [39,40], the homogeneity hypothesis in the existing literature, whether for studying the economic impact of fertilizer use or econometric models at other levels, denies the impact of various economic structures, resource endowments, locational conditions, technology markets, infrastructure, and government policies in different regions [41]. If the effect of fertilizer use on AEE is tested based on the homogeneity hypothesis, this may lead to biased estimation of results and limitations in their application [42]. Therefore, it is crucial to consider heterogeneity among regions.

Since heterogeneity is not investigated in most existing studies, this paper intends to construct a long-term panel quantile regression model (QRM) using inter-provincial long-run panel data in China from 1978–2020. This is to examine the effect of fertilizer use intensity (FUI) on agricultural eco-efficiency (AEE) in heterogeneous conditions and to explore the differences and changes in the impact of FUI in different economic sub-divisions and sub-periods. With the improvement of agricultural market economy and the expansion of inter-regional openness, the spatial mobility of agricultural production factors is becoming more frequent, and the spatial link between agricultural production is getting closer [43,44], leading to the spillover effect of fertilizer use on a spatial scale. Hence, it is equally crucial to consider the spatial effect between regions. This paper also introduces the spatial lag factor into the econometric model to further examine the impact of fertilizer use under the spatial interaction effect.

## 3. Materials and Methods

### 3.1. Super-Efficient SBM Model

During agricultural production, the optimum outcome is greater economic production generated by input factors accompanied by less environmental pollution due to the excessive use of chemicals such as fertilizer, pesticides, and agricultural films. The former is called the desired output, and the latter, non-desired. Therefore, the measurement of agricultural eco-efficiency (AEE) should consider both the desired and non-desired outputs.

The slacks-based measure (SBM) model based on non-desired output first proposed by Tone [45] was adopted to measure eco-efficiency. Compared with the traditional data envelopment model (DEA), the SBM model can effectively solve the “crowding” or “slack” phenomenon of input factors caused by the radial and angular traditional DEA model. Still, like the conventional DEA model, for DMUs (decision-making unit) with efficiency = 1, it is also difficult to distinguish the efficiency difference between efficient DMUs. Tone [46] defines the super-efficient SBM model based on the basic SBM model: the mix of the merits of the super-efficient DEA model and the basic SBM one, enables it to carry out effective yet further comparison and evaluation of the DMUs on the frontier. Therefore, in this paper, the super-efficient SBM model was used to measure AEE, and the specific model is constructed as follows:

(1) Min ρ=1m∑i=1mx¯xik1r1+r2∑s=1r1yd¯/yskd+∑q=1r2yu¯/yqku

(2) $$\left\{\begin{array}{l} \bar{x}\geq \sum_{j=1,\neq k}^{n}x_{ij}\lambda_{j};\bar{y^{d}}\leq \sum_{j=1,\neq k}^{n}y_{sj}^{d}\lambda_{j};\bar{y^{d}}\geq \sum_{j=1,\neq k}^{n}y_{qj}^{d}\lambda_{j};\\ \bar{x}\geq x_{k};\bar{y^{d}}\leq y_{k}^{d};\bar{y^{u}}\geq y_{k}^{u}\\ \lambda_{j}\geq 0,i=1,2,\ldots ,m;j=1,2,\ldots ,n,j\neq 0;\\ s=1,2,\ldots ,r_{1};q=1,2,\ldots ,r_{2}; \end{array}\right.$$

In the formula, it is assumed that there are *n* DMUs with each DMU consisting of input (*m*), desired output (*r*_1_), non-desired output (*r*_2_), *x*, *y^d^*, *y^u^*, the element of the input matrix, desired output matrix and non-desired output matrix, respectively, and *ρ*, the value of AEE.

The agriculture in this paper refers to the planting industry in a narrow sense. Referring to the relevant literature [19,47], combined with data availability and statistical caliber consistency, the evaluation index system of AEE was constructed according to the inputs, agricultural outputs, and ecological environmental impact in the agricultural production process, with land, labor, mechanical power, irrigation, chemical fertilizer, and pesticides taken as input indicators, the total agricultural output value as desired outputs, and agricultural non-point source pollution emissions as non-desired outputs (Table 1).

Next, we need to measure the level of non-desired outputs [47,48]. Agricultural non-point source pollution is mainly caused by the excessive use of fertilizer, pesticides, and agricultural films. In this paper, the sum of fertilizer loss, ineffective pesticide utilization, and agricultural film residue was used to estimate the level of agricultural non-point source pollution [47]. (1) The pollutant indicators for fertilizer loss calculation are total nitrogen (TN) and total phosphorus (TP), which are stratified into three types: nitrogen fertilizer, phosphate fertilizer, and compound fertilizer. The emission coefficient of pollution units is equal to the pollution production coefficient multiplied by the fertilizer loss rate. The TN pollution production coefficients of nitrogen, phosphate, and compound fertilizer are 1.0 and 0.33, and the TP pollution production coefficients are 0, 0.44, and 0.15, respectively [29]. The fertilizer loss rate in each region was taken from the study of Lai et al. [49]. Fertilizer loss is the amount of nitrogen, phosphate, and compound fertilizer use × emission coefficient. (2) The formula for calculating ineffective pesticide utilization was pesticide use amount × ineffective pesticide utilization coefficient, with that of agricultural film residues being agricultural film use amount × agricultural film residue coefficient. The above two coefficients of pollution emissions are taken from Wu et al. [50] and “Manual of First National Pollution Census: Pesticide Loss Coefficient and Agricultural Film Residue Coefficient”, with regional cultivated land topography differences.

### 3.2. Baseline Model and Variable Selection

To empirically test the impact of FUI on AEE, a baseline econometric model was first constructed based on the Stochastic Impacts by Regression on Population, Affluence and Technology model (STIRPAT, *I* = *aP^b^A^c^T^d^e*), usually in its logarithmic form: ln*I* = ln*a* + *b*ln*P* + *c*ln*A* + *d*ln*T* + ln*e*, where *P*, *A*, and *T* denote population, affluence, and technology, respectively, *b*, *c*, and *d* are their elasticity coefficients, and *a* is a constant term [51]. In this paper, the variable is AEE and the core explanatory variable is FUI. We use rural labor transfer (RLT) to reflect population changes, per capita disposable income of rural households (DIR) to reflect affluence level, mechanical input intensity (MII) to reflect the technologal level, and introduce other structural factors such as the multiple crop index (MCI), crop planting structure (CPS), and fiscal supporting on agriculture (FSA) to extend the STIRPAT model.

(3) $$\ln{\mathrm{AEE}}_{\mathrm{it}}=\beta_{0}+\beta_{1}\ln{\mathrm{FUI}}_{\mathrm{it}}+\beta_{2}\ln{\mathrm{RLT}}_{\mathrm{it}}+\beta_{3}\ln{\mathrm{DIR}}_{\mathrm{it}}+\beta_{4}\ln{\mathrm{MII}}_{\mathrm{it}}+\beta_{5}\ln{\mathrm{MCI}}_{\mathrm{it}}+\beta_{6}\ln{\mathrm{CPS}}_{\mathrm{it}}+\beta_{7}\ln{\mathrm{FSA}}_{\mathrm{it}}+\varepsilon_{\mathrm{it}}$$

In Equation (3), in terms of variable selection, AEE is the agricultural eco-efficiency measured based on the SBM model, FUI is characterized by the fertilizer use per unit of crop sown area, and FUI = agricultural fertilizer use (discounted pure amount)/total crop sown area (kg/hm^2^). *ε_it_* represents the residual term. The other variables are specified as:

(i). Rural labor transfer (RLT) = rural employees-agricultural employees. The difficult data acquisition of provincial rural transfer from existing statistics means this paper can only define it from an employment standpoint. The rural labor transfer direction from the agricultural to the non-agricultural realm coincides with its changed number [15]. The index explanation part of the China Statistical Yearbook shows that rural employees are divided into agriculture, industry, construction, and other fields. According to the longest time engaged in the main sector (the same time according to income). The real situation of rural employees in the non-agricultural industries agrees with the definition in this paper.
(ii). Disposable income of rural households per capita (DIR). The affluence of rural households that represent the basis for agricultural production and management can influence the scale and structure of agricultural input factors affecting AEE characterized by the per capita disposable income of rural households.
(iii). Machinery input intensity (MII) = total power of agricultural machinery/per unit sown area. At present, China’s agricultural production techniques have completed the transformation from relying mainly on human and animal power to relying mainly on mechanical power. Machinery use, the most intuitive manifestation of the agricultural technology progress, can improve agricultural output by popularizing machinery services and promoting labor productivity by effectively replacing the labor force.
(iv). Multiple crop index (MCI) = total crop sown area/cropland area. Multiple crop index, the average number of crops planted on the same cultivated land in a certain period (usually one year), is adopted to reflect the impact of changes in the degree of cultivated land use on AEE. The proportion of sown area to cropland area is usually used to characterize the MCI, where the sown area is similar to the gross cropped area and cultivated land area is similar to the net cropped area.
(v). Crop planting structure (CPS) = grain crop planting area/total crop sown area. Planting structure refers to the proportion of crop types grown in a certain area with its change leading to the changed agricultural input factor structure, affecting AEE.
(vi). Fiscal supporting on agriculture (FSA) = fiscal expenditure on agriculture, forestry, and water affairs/total crop sown area. The subsidy intensity of financial funds to agriculture can affect the input of rural residents to agricultural resources such as chemical fertilizer, pesticides, and agricultural machinery services and can reflect the impact of the administrative intervention on AEE.

### 3.3. Quantile Regression Model

The traditional panel regression model, also the mean regression model, has parameter values estimating conditional expectations for the dependent variable, only reflecting the average marginal impact of the independent variable on the expectations of the dependent counterpart [52]. However, for regions with different resource endowment bases we see the various impacts of FUI on AEE. The advantages of the quantile regression model (QRM) are that it is a separated model for different quantile points and its full capture of conditional distribution skewness. The QRM, first proposed by Koenker in 1978 [53], is an extended linear model for regressing independent variables based on the conditional quantile of dependent variables. Compared with the ordinary OLS model, QRM is less influenced by outliers of dependent variables than mean regression without assumptions about the distribution of random disturbance terms. Its selection of arbitrary quartiles of the dependent variable for parameter estimation can minimize extreme values in the estimation results [54]. So, we further constructed a panel QRM based on the baseline model to examine in depth the heterogeneity of FUI impacts. QRM specifically estimates the elasticity coefficients of conditional distribution Y/X in terms of several quantile points across the board and uses a weighted average of the absolute values of residuals as the objective minimization function to eliminate estimation errors due to outliers.

With the development of panel econometric models, Koenker [55] extended quantile regression to panel data, and the *θ* quantile of explanatory variable Y was defined as:

(4) $$Quant_{\theta }(Y_{it}\left|Z_{it}\right.)=\inf\left\{y:F_{Y|Z}(y)\geq \theta \right\},0<\theta <1$$

Then, the QRM can be expressed as:

(5) $$Quant_{\theta }(Y_{it}\left|Z_{it}\right.)=Z_{it}^{T}\beta_{\theta }+\varepsilon_{\theta }$$

In the formula, *Y_it_* is the AEE, *Z_it_* is the influencing factor of AEE, $Quant_{\theta }(Y_{it}\left|Z_{it}\right.)$ is the conditional quantile of *Y_it_* corresponding to quantile *θ* (0 < *θ* < 1) for a given *Z_it_*, *ε_θ_* is the residual vector, and *β_θ_* is the coefficient to be estimated for *θ* quantile, whose estimator is obtained by minimizing the asymmetric weighted absolute outlier sum [56]. When $Y_{it}{\;>\;Z}_{it}^{T}\beta_{\theta }$, the weight of absolute deviation is *θ*, while $Y_{it}{\;<\;Z}_{it}^{T}\beta_{\theta }$, the weight of absolute deviation is (1 − *θ*):

(6) $$\beta_{\theta }=\underset{\beta_{\theta }}{\min}\left\{\theta \sum_{i,t:Y_{it}\geq Z_{it}^{T}\beta_{\theta }}^{n}\left|Y_{it}-Z_{it}^{T}\beta_{\theta }\right|+(1-\theta )\sum_{i,t:Y_{it}<Z_{it}^{T}\beta_{\theta }}^{n}\left|Y_{it}-Z_{it}^{T}\beta_{\theta }\right|\right\}$$

### 3.4. Data Sources

Agriculture in the broad sense includes agriculture, animal husbandry, and fishery, while in the narrow sense means crop planting, so the empirical evidence in this paper focuses on agriculture in the narrow sense. The research focusses on 30 provinces (municipalities and autonomous regions) in China but does not include Tibet, Hong Kong, Macao, and Taiwan given their special resource endowment, agricultural production conditions, and data availability. The required basic data were obtained from the China Rural Statistical Yearbook, China Agricultural Statistics, Agricultural Statistics of New China in the Past Fifty Years, and the National Bureau of Statistics data website, available online: http://data.stats.gov.cn/easyquery.htm?cn=E0103 (accessed on 25 March 2022). Some of the missing data were obtained by consulting the provincial statistical yearbooks or 60-year statistical data, with still missing data filled by interpolation. The data of Chongqing before 1997 and Hainan before 1988 were obtained through their respective statistical yearbooks and 60-year statistical data and adjusted to the corresponding data of Sichuan and Guangdong. This allowed the panel data of 30 provinces for 43 years from 1978 to 2020 to be finalized. Table 2 shows the descriptive statistics for each variable.

## 4. Results

### 4.1. Measurement of AEE

**Figure 1:**
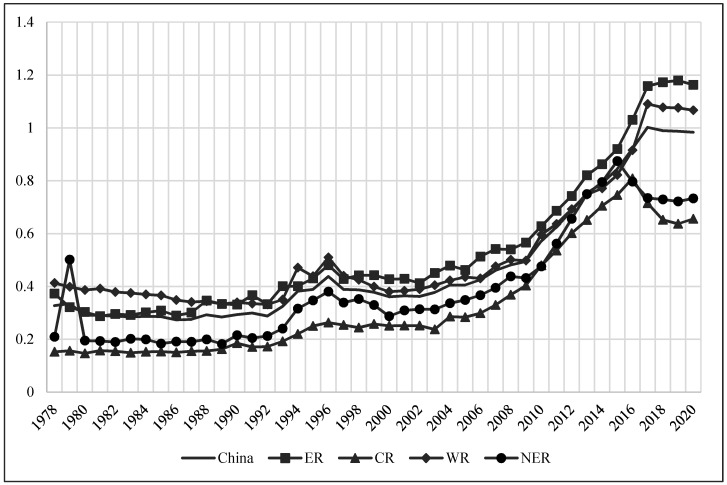
Evolution of AEE in China from 1978–2020. The reference for economic division is available online: http://www.stats.gov.cn/ztjc/zthd/sjtjr/dejtjkfr/tjkp/201106/t20110613_71947.htm (accessed on 25 March 2022), which contains 10 provinces in the ER, including Beijing, Tianjin, Hebei, Shanghai, Jiangsu, Zhejiang, Fujian, Shandong, Guangdong and Hainan; 6 provinces in the CR, including Shanxi, Anhui, Jiangxi, Henan, Hubei and Hunan; 12 provinces in the WR, including Inner Mongolia, Guangxi, Chongqing, Sichuan, Guizhou, Yunnan, Tibet, Shaanxi, Gansu, Qinghai, Ningxia and Xinjiang; and 3 provinces in the NER, including Liaoning, Jilin and Heilongjiang (Tibet, Hong Kong, Macao and Taiwan are not included in this study).

After having measured the AEE of 30 provinces in China from 1978–2020, the super-efficient SBM model based on non-radial (non-Oriented) variable returns to scale (VRS) solved for the mean value of each year, and also divided the country into four major regions according to economic subdivisions: Eastern region (ER), Central region (CR), Western region (WR) and Northeastern region (NER), to compare and analyze the mean values of AEE in different regions (Figure 1).

(1) The interpretation of the trend reveals that the average AEE, which is essentially under 0.8 in most years, sees a low level of AEE with a stable upward trend in China. However, the years 1978–2000 saw volatility and fluctuation in AEE with a small increase magnitude, while from the year 2000 onwards it enjoyed a stable upward trend, before falling back after 2016.
(2) The division into two stages with the year 2000 as the boundary in the context of comparing the AEE of the four major regions. The ranking of AEE during 1978–2000 is WR > ER > NER > CR, while during 2000–2018 is ER > WR > NER > CR, with a small gap between the CR and the WR, while after 2016, the change of AEE in the NER and CR is the opposite trend from the ER and WR, with the gap between regions first widening and then narrowing.

**Figure 2:**
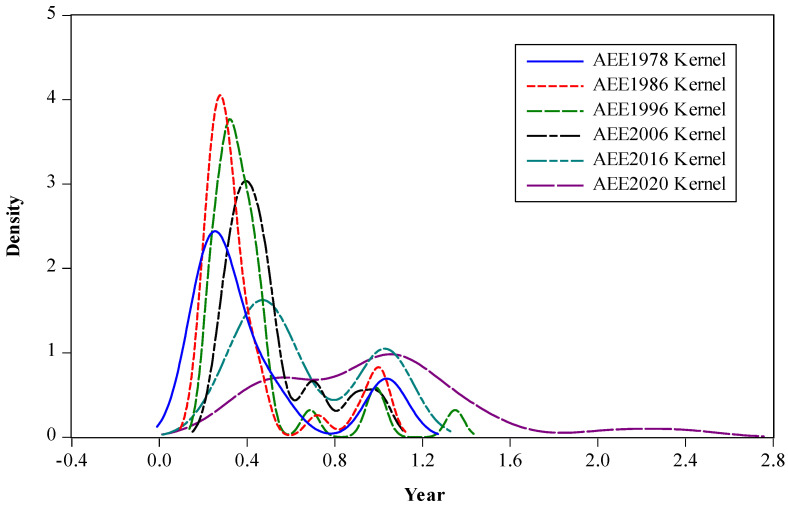
Kernel density estimation of AEE in China.

To continue to analyze the clustering differences in the evolution of AEE over time, a non-parametric kernel density function with a Gaussian normal distribution was adopted [57], and the six years of 1978, 1986, 1996, 2006, 2016, and 2020 were selected as observation time points for kernel density estimation (KDE). The wave crest height reflects the agglomeration degree of AEE in each province (Figure 2). The overall distribution of AEE in China shows a “bimodal” evolution from left to right, with peaks ranging from high to low, indicating that China’s AEE increases steadily with time, with most provinces gradually shifting from low-level agglomeration to a narrowing trend of “high–low” discrete differences.

At the beginning of reform and opening up, the AEE of most provinces concentrated at a low level. After the 1990s, with the enhancement of agricultural environmental protection consciousness and the acceleration of the agricultural mechanization process, the AEE of each province was improved to varying degrees. However, due to the differences in resource endowment and economic strength between provinces, the gap in AEE between provinces began to increase, forming several peaks of different magnitudes, and the trend of low agglomeration gradually decreased. By 2016, the wave height of bimodal distribution narrowed, the gap in AEE was further decreased, and a near “bimodal” evolution pattern of “low-low and high-high agglomeration” was essentially formed. But after 2016, influenced by agricultural endowment, production cost, and environmental protection pressure, the gap in AEE between regions expanded, and the overall distribution was unimodal.

In the 21st century, the No. 1 Document of the Central Committee has focused on agriculture for many years, paying attention to the issues of agriculture, rural areas and farmers, and proposing the idea that “we should encourage the development of circular agriculture and ecological agriculture.” It shows that the government attaches importance to sustainable agricultural development to prohibit AEE from effectively declining. Given the low AEE situation, there is much room for achieving resource conservation and environmental protection in the sustainable development of agriculture in China [19]. In addition, the regional differences in AEE mainly stem from the fact that, with the continuous development of the agricultural economy, the differences in agricultural mechanization and technological progress are gradually highlighted between regions due to objective factors such as development level and terrain conditions. For example, ER has fast development in the agricultural economy, owing to its advanced agricultural technology, more attention to agricultural modernization, and the coordination between agricultural production, resource conservation, and environmental protection. On the contrary, CR is affected by the hilly terrain, has relatively slow development in agricultural technology, a low degree of agricultural mechanization, and the development of agricultural production still needs further adjustment.

### 4.2. The Heterogeneity Impact of FUI on AEE

To avoid pseudo-regression problems, unit root tests on the panel data must ensure the stationarity of variables. In this paper, the tests were conducted with four test methods, LLC (Levine-Lin-Chu), IPS (Im-Pesaran-Skin), ADF-Fisher, and Harris-Tzavalis (Table 3). The results showed that although individual variables fail the significance test under certain methods, considered together, it can be concluded that rejecting the original hypothesis that there were unit roots in the variables is correct, and the variables can be considered stationary. In addition, the variance inflation factors (VIF) of the independent variables are all significantly less than 10, with an average VIF of 2.87, indicating that there is no significant multicollinearity problem among the variables.

The Hausman test results favored a panel QRM with a fixed effect. Table 4 shows the estimation results of the QRM for the full sample, and the baseline panel econometric result was added as a reference to observe the average treatment effect of FUI (Table 4). The baseline regression result showed that FUI had a significant negative effect on AEE.

**Figure 3:**
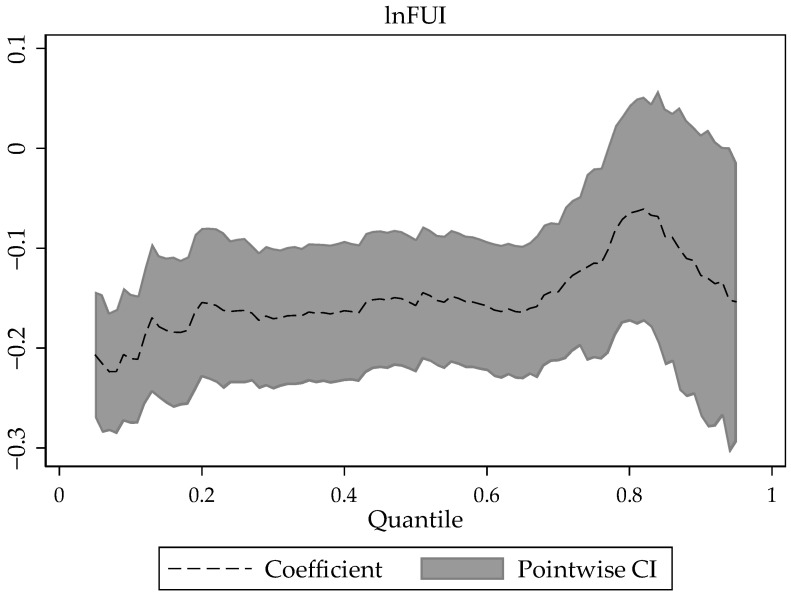
The trend of the estimated coefficient of FUI.

The panel QRM is estimated using 10 quantile points. The coefficients of FUI are all significantly negative, indicating that FUI has had a significant inhibitory effect on AEE at the provincial level since the reform and opening up, and that overuse of fertilizer is not conducive to AEE, consistent with the result of the baseline regression result. Each 1% increase in FUI can lead to a decrease in AEE in the range of [−0.211, −0.065] (Figure 3), with the increase of the quantile, the negative coefficient of FUI shows a steady upward trend of rising first and then falling, that is, the negative effect of FUI on AEE shows a gradual weakening and then a slight rebounding, with a high negative effect at the low quantile, and the smallest negative effect on AEE when FUI is at the 80% quantile.

For regions with a lower level of AEE, increasing the FUI can lead to a higher inhibitory effect on the AEE. Areas with low efficiency where more attention has been paid to the economic benefits from agricultural production, the ideal equilibrium among inputs, economic benefits, and ecological impacts has not yet been reached, and the cost and benefits are not enough to support the efficiency improvement in these areas; thus, excessive fertilizer use has a relatively large inhibitory effect on the AEE [19]. While for regions with a higher level of AEE, the negative impact of FUI tends to be weaker, though slightly rebounding. We believe that the inputs, agricultural output, and ecological environment impact in higher efficiency areas can achieve an equilibrium, and can effectively match the reasonable scale and intensity of fertilizer use [58].

From the coefficients of the control variables,

(1) RLT has a inhibitory effect on the AEE, and this negative impact shows a persistent enhancement as the quantile rises, indicating that areas with a higher AEE are more negatively affected by rural labor transfer.
(2) The increase of DIR helps improve AEE, which is similar to the findings of Xu et al. [59]. In addition, the positive impact of DIR increases with the increase of the quantile, indicating that areas with higher AEE are more positively affected by the DIR.
(3) The coefficients of MII showed a shift from negative to positive at different quantile points, indicating that the negative effect of MII on the AEE gradually disappeared and the positive effect gradually appeared as the quantile points rose. However, the impact of MII was not significant at the low quantile (10%, 20%) and high quantile (90%), implying that areas with low and high AEE were not significantly affected by machinery inputs.
(4) The coefficient changes of the MCI at different quantile points were similar to those of the MII, which also showed a shift from negative to positive, starting from the 30% quantile to be significantly positive, and reaching the maximum at the 80% quantile, indicating that the positive effect of the MCI gradually increased as the quantile point rose, and that the positive effect of the MCI was more prominent in areas with a high AEE.
(5) The effect of CPS on the AEE was significantly negative at different quantile points, and this negative impact showed a continuous enhancement of transformation characteristics as the quantile point increased, reaching the highest at the 90% quantile, implying that the increase in the proportion of food crop cultivation is not conducive to enhancing AEE, and the areas with higher AEE are more negatively affected by the CPS.
(6) The coefficients of FAS also showed a shift from negative to positive at different quartiles, with negative coefficients gradually insignificant and positive coefficients gradually significant; and the positive effect reached the highest point at the 90% quartile, indicating that FSA had a stronger positive promoting effect on the more efficient regions and a more negative inhibiting effect on the less efficient regions.

### 4.3. Spatial–Temporal Differences of FUI Impact

The differences in the effects of FUI on AEE were further examined in different regions and periods (Table 5). Firstly, considering the spatial variation of provincial samples, the spatial heterogeneity of the impact of FUI on the AEE was investigated according to different economic sub-divisions. Secondly, China started a large-scale comprehensive agricultural development in 1988 to promote sustainable development in agriculture. In addition, since 2004, the central government has issued the No. 1 Document on the “agriculture, rural areas and farmers” and completely abolished the agricultural tax in 2006. Considering the historical nodes of agricultural policies and the time lag of policy effects, the development of China’s agricultural economy since the reform and opening up can be divided into three periods: 1978–1989, 1990–2003, and 2004–2020, and the temporal heterogeneity of the impact of FUI can be examined.

**Figure A1:**
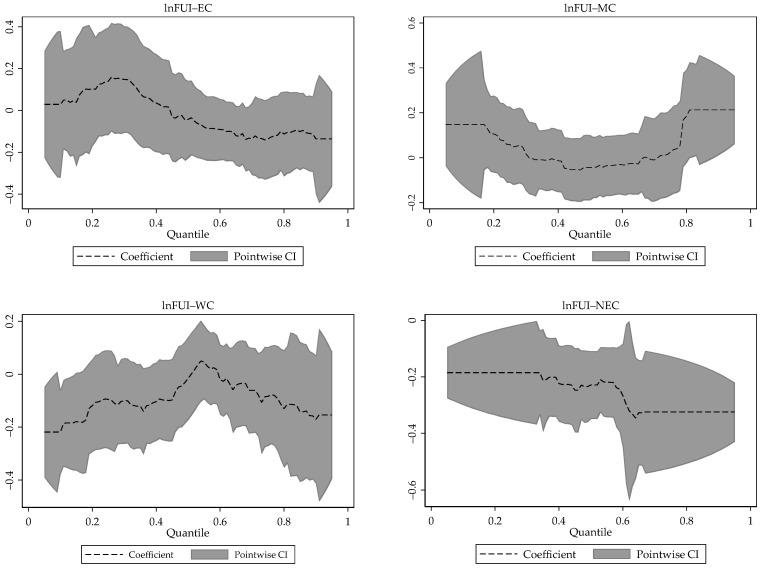
Variation of FUI coefficients in economic subdivisions at different quartiles.

From the results of different grouping criteria, FUI showed different impacts between regions in different economic sub-divisions, and there is a need to consider spatial heterogeneity. The baseline regression results show that the coefficients of FUI were significantly negative in all regions, and that the negative effect of average treatment was greatest in the ER, followed by the CR, indicating that reducing the intensity of fertilizer use in these regions is beneficial to improving the local AEE. The quantile regression results showed that the coefficients were significantly negative at most of the quantiles in all regions, which overall, indicated that FUI had a negative effect on the AEE, reflecting the robustness of the conclusions side-by-side. However, the coefficients of FUI in different regions showed different variation characteristics with the increase of quantiles (Figure A1 in Appendix A). As the quantile rises:

(1) The coefficients in the ER undergo a similar U-shaped change from positive to negative, strengthening and then weakening, with the coefficient turning from positive to negative in 40~50% quantile, and the greatest positive impact at the 30% quantile and the strongest negative impact at the 90% quantile. This indicates that the FUI has a positive promoting effect on the lower AEE regions and a negative inhibiting effect on the more efficient regions in the ER, where the greater the quartile, the stronger the negative impact, which is inconsistent with the results at the national level and may be related to the differences in endowment conditions and the positioning of agricultural production within the ER.
(2) The coefficients in the CR showed a transformation from “positive → negative → positive”, first weakening negatively and then strengthening positively, with a negative effect in 40~70% quartiles and a positive effect at both lower and higher quartiles. This indicates that FUI has a significant positive effect on both the lower and higher AEE areas in the CR, but does not have a significant negative effect on the areas with intermediate efficiency, probably because the provinces in the CR are mainly grain-producing areas with better agricultural production conditions, and fertilizer use tends to be dynamically balanced.
(3) The coefficients in the WR showed a similar inverted U-shaped shift of rising and then falling, except for a weaker but insignificant negative effect at the 50~70% quantile, and the significantly negative effect at all other quantiles with larger negative inhibition at both the low and high quartiles. This indicates that FUI has a stronger negative inhibitory effect in the WR regions with lower and higher agroecological efficiency. This is closely related to the agricultural production conditions and endowment base in the WR, and for the WR regions with high efficiency, increasing FUI is more likely to break the equilibrium relationship between inputs and outputs.
(4) The coefficients in the NER were all significantly negative and gradually strengthening, but the negative effect was more stable at both the low and high quartiles, indicating that FUI has a significant negative effect on areas in the NER at different efficiency levels, and the higher the AEE, the more pronounced the negative inhibitory effect of FUI.

**Figure A2:**
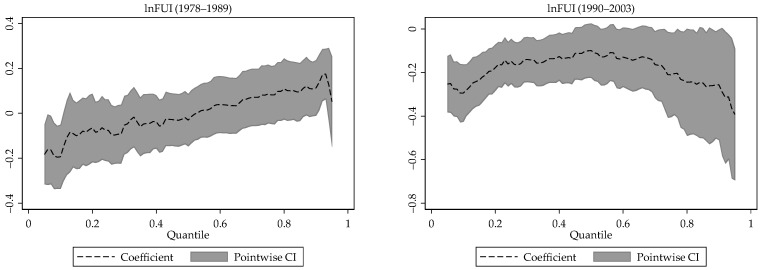

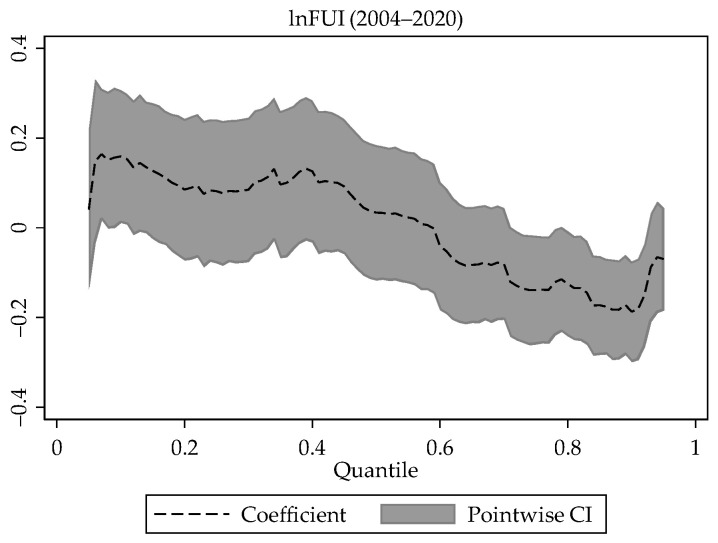
Variation of FUI coefficients in sub-periods at different quartiles.

From the results of the different period groupings, the baseline regression results show that FUI had a significant negative impact on the AEE only during 1990–2003, but the quantile regression results show that the impact and significance levels of FUI at different quantile points within each period showed variability (Figure A2 in Appendix A).

(1) The FUI coefficients during 1978–1989 show a negative to positive shift at different quartiles, with the negative effect weakening and positive effect increasing and starting to exert positive effect after the 60% quantile but only reaching significance at the high (80~90%) quantile, where fertilizer has a positive enhancing effect. This indicates that FUI had a negative inhibitory effect on areas with low AEE, and a positive promoting effect on areas with high AEE during this period.
(2) The FUI coefficients during 1993–2003 were significantly negative at all quartiles, and this negative effect showed a first weakening and then strengthening shift, with a less negative effect of FUI on areas at the 40% to 60% quartiles; indicating that FUI had a strong negative inhibitory effect on both high and low AEE areas during this period.
(3) The FUI coefficients during 2004–2020 showed a shift from positive to negative at each quantile, with the positive effect weakening and negative effect increasing, which is the opposite of the period 1978–1989, when FUI had a stronger negative effect on areas in the high quantile (80~90%).

### 4.4. Considering the Spatial Effect of Agricultural Production

Agricultural production is not isolated in space, and there is heterogeneity. The economic phenomenon in any region has a certain correlation with its surrounding areas, and the closer the geographical distance is, the closer the correlation between regions [60]. If the spatial correlation between regions is ignored, the estimation results may be biased and inconsistent [61]. Therefore, spatial effect needs to be introduced into the QRM [62], so, the spatial lag term of FUI is added to the QRM to construct a spatial quantile regression model (SQRM) to investigate the spatial differences and variations in the influence of FUIs.

We have constructed three different forms of spatial weight matrices: (i) Rook adjacency matrix (*Wq*), which is set based on the Rook spatial adjacency relationship; (ii) geographical distance matrix (*Wd*), in which the elements take values as the inverse of Euclidean distance between the capital cities of the two provinces [63]; (iii) agricultural economic distance matrix (*Wa*), in which the elements take values as the product of the geographic distance weight matrix (*Wd*) and the diagonal matrix of agricultural economic scale [64]. The above three weight matrices are all row standardization.

The distribution range of Moran’s I of the three spatial weight matrices is 0.1917~0.4598, and all of them passed the significance test, indicating that there is a significant positive correlation of FUI in space, so it is feasible to introduce a spatial lag term into the QRM (Table 6). Only the estimated coefficients of FUI and its spatial lag term are reported.

The regression results show that, under the premise of considering different spatial weight matrices, the coefficients of FUI had a transformation process in which the negative effect gradually decreases with the rise of quantile. Compared with the result of the ordinary QRM, the transformation process of negative coefficients is more similar at different quantiles, but the decay rate of negative effects is faster under different spatial weight matrices. Under *Wd* and *Wa*, the coefficient of FUI shows a transition from negative to positive, which means the existence of spatial effects; there is a more obvious strategic imitative interaction for areas with higher levels of AEE, and the demonstration effect and driving effect generated by spatial linkage promote the communication and exchange of production factors and utilization methods between regions and helps to dissipate the negative effects brought by fertilizer use.

The coefficients of the spatial lag term of FUI, except for the negative to positive shift under *Wq*, were significantly positive under both *Wd* and *Wa*, which took into account geographical distance and economic level, indicating overall that FUI had a negative impact on the local AEE, but a positive spillover effect on the AEE of neighboring areas, mainly due to the feedback interaction effect among neighboring areas [65]. The positive spillover effect of FUI is stronger under *Wd*, and after superimposing economic factors (*Wa*), this spillover effect decreases, which may be because the agricultural economic distance weight matrix takes into account not only the influence generated by the geographical distance between regions but also the difference in agricultural economic scale, which has a dragging role on this positive spillover effect of FUI. In addition, with the increase of the quantile, the positive spillover effect of FUI gradually increased, implying that the areas with higher AEE would have a stronger positive spatial spillover effect.

## 5. Discussion

Since the reform and opening up, great changes have taken place in developing the agricultural economy in China. Analyzing the data of agricultural inputs, we find that the rural labor force in most provinces shows a downward trend, while the material factors of production, such as machinery services and the use of fertilizers, show an upward trend to varying degrees, which leads to a change in stable crop sowing area. In addition, with the urbanization process and the massive outflow of rural labor, the development of China’s agricultural economy has changed from a stage where labor and material factors of production such as mechanization were “complementary”, to a stage where material factors such as mechanization are “substitutes” for labor. However, the ongoing substitution process has increased the expected output of agriculture and increased the agricultural non-point source of pollution.

This study found that the negative effect of FUI on the AEE was robust in an average sense, but there were differences in the effect of FUI for areas at different levels of efficiency. Agricultural production plays an essential role in socioeconomic development; however, agricultural development can be unsustainable due to the negative environmental effects of pollutant inputs such as fertilizers [66], so it is worthwhile to study the impact of FUI on AEE. However, agricultural production varies significantly among regions regarding resource endowment, technical equipment, development level and structure, infrastructure, and other aspects. Comparing the results of the baseline regression with the QRM, the baseline regression can only reflect the impact of FUI on the AEE in an average sense, masking the heterogeneous effect caused by differences in regional development. Ordinary panel regression models based on the homogeneity hypothesis may yield biased conclusions, whereas the QRM can account for the differential changes in the impact of FUI among different regions in detail, so it is necessary to consider the conditions of inter-regional heterogeneity in the impact of FUI on AEE [64].

Currently, China is facing the problems of inefficient fertilizer use and pressure to increase grain production. It is helpful to strengthen the management of the structure, method, and intensity of chemical fertilizer use, which could enhance grain production and reduce the environmental pollution of farmland caused by excessive fertilizer use [67]. Considering the goal of zero growth in fertilizer use, there are still unmet needs in different regions and crops, resulting in regional heterogeneity in the impact of chemical fertilizer use on the AEE, so regions with different levels of AEE should follow the principle of adapting to local conditions and set different fertilizer reduction standards.

The Chinese government has realized the dual threats to agricultural production and farmland environment caused by excessive fertilizer input and has issued the “Action Plan for Zero Growth in Fertilizer Use”, which aspires to maintain fertilizer use in major crops at the current level through scientific fertilization management and technical systems. Fertilizer use is the major source of agricultural carbon emissions [24,68], the reduction and zero growth of fertilizer are key to ensuring the effective supply of agricultural products and promoting the green development of agricultural production. Fertilizer zero growth is a dynamic goal, which does not mean no input of chemical fertilizer during crop production, but rather to improve fertilizer use efficiency and agricultural productivity by changing fertilizer utilization methods and reducing unreasonable use of fertilizer. Fertilizer reduction belongs to process management, which means not only a decline in the scale of use but also the intensity of fertilizer use must be controlled at reasonable levels. It is also important to note that there are differences in growth conditions in different regions, and differences in fertilizer demand for the growth of different crops, so, the reduction action should focus on local conditions and be crop specific while still ensuring the necessary output capacity.

Around the world, countries are also facing the problems of non-point source pollution and efficiency reduction caused by excessive fertilizer use. Although the agricultural production patterns in various countries are quite different, China’s government-led and farmer-involved approach to fertilizer reduction can provide lessons for other countries that also need to control excessive fertilizer input according to their own national conditions. For example, India, a major agricultural country in South Asia, is promoting a plan to reduce its fertilizer use by at least 10%, but the implementation of the plan requires a heterogeneous fertilizer reduction program based on the endowment differences between different regions of the country [69] and guides the public to participate in order to reduce the impact of fertilizer on the agroecology and the loss of efficiency.

Although this paper has obtained interesting conclusions, there is still room for improvement. For example, we only reflected agricultural technological progress through the popularization of mechanization, but the use of technological innovations in seed varieties and quality in agricultural production was limited by the difficulty of characterization and macro data acquisition and so are not reflected in our study. In future research, methods to scientifically and rationally assess agricultural technological progress requires our focus.

## 6. Conclusions

In this paper, a panel QRM has been constructed to test the effect of FUI on the AEE at the provincial level based on the heterogeneous conditions, and the differences of this effect in different regions and time periods has been explored. The spatial lag term of FUI has been further introduced to discuss the changes in the effect on AEE under the spatial effect.

(1) AEE of China shows a stable upward trend amidst fluctuations, remains at a low level overall, and there is more room for resource conservation and environmental protection, with significant inter-regional differences. Fluctuation in the AEE is mainly concentrated between 1978–2000, with a more pronounced increase in the eastern regions than in the mid-west and western regions during 2001–2020.
(2) At different quantiles, the increase in FUI has a significant negative inhibitory effect on the improvement of AEE. The negative effect of FUI on AEE showed a gradual weakening and then a slight rebound shift as the quantile increased. FUI had a stronger negative effect at the lower quantile.
(3) For different economic sub-divisions, FUI in the ER had a positive promoting effect on areas with lower AEE, FUI in the CR had a significant positive effect on areas of both lower and higher AEE, and FUI in the WR had a stronger negative inhibiting effect on areas of both lower and higher AEE, and FUI in the northeast region has a more pronounced negative inhibiting effect on areas of higher AEE. For different sub-periods, FUI positively promoted the areas with high AEE in 1978–1989, then FUI had a strong negative inhibitory effect on areas of both high and low AEE in 1993–2003, and FUI had a stronger negative effect on areas of high AEE in 2004–2020.
(4) After considering the spatial lag of FUI, the negative effect of FUI undergoes a gradual decreasing transformation with the rise of quantile, and the decay of this negative effect was faster. Although FUI had a negative effect on local AEE, it had a positive spillover effect on the AEE of neighboring areas, and the higher the AEE, the stronger the positive spatial spillover effect of fertilizer use.

## 7. Policy Implications

Reducing the intensity of fertilizer use and negative environmental externalities, and improving fertilizer use efficiency have become the consensus of the whole of society. The policy implication of this paper is that considering heterogeneous conditions in different regions can help provide a more differentiated explanation for the effects of fertilizer use intensity on agricultural eco-efficiency. The impact of fertilizer use varies significantly across regions, and time periods, all of which indicate that the central and local governments should dynamically adjust the fertilizer reduction and efficiency policies according to the development of the AEE in different regions, depending on local conditions.

On the one hand, government departments should focus on the sustainable growth goal of serving “two types of agriculture” (resource-saving and environment-friendly) and zero growth in fertilizer use, make differentiated deployment according to the heterogeneous conditions of agricultural production, dynamically adjust the input structure of agricultural production factors, reasonably control the proportion of rural labor transfer, optimize crop planting structure, continue to increase financial support to agriculture, and strengthen agricultural science and technology research while pursuing the improvement of agricultural economic benefits. It is also essential to restrain the excessive growth of fertilizer use per unit area and substitute mineral or chemical fertilizers with mineral or organic fertilizers to enrich soil fertility and maintain farmland productivity [70]. On the other hand, neighboring regions should be given more emphasis because the fact has been confirmed that negative effects can be influenced by the spatial effect of fertilizer use in neighboring regions. Therefore, while paying attention to the local fertilizer reduction and efficiency improvement, neighboring regions should also establish a complete agro-ecological cooperation mechanism, improve inter-regional fertilizer reduction and efficiency improvement policies, and strengthen agricultural production cooperation and exchange, which would be of great practical significance to ensure national food security and ecological safety.

## Figures and Tables

**Table 1:** Evaluation index system of AEE.

Indicators	Variables	Variable Description	Remarks
Elemental inputs (*x*)	Land	Total crop sown area/khm^2^	Reflects the actual cultivated area in agricultural production
	Labor	Agricultural employees/10^4^ people	Primary industry employees × (Gross agricultural product/Gross output of agriculture, forestry, animal husbandry and fishery)
	Mechanical	Total power of machinery/10^4^ kW	Agricultural machinery is a representative tool of agricultural modernization
	Water	Effective irrigation area/khm^2^	To characterize the irrigation level of agricultural water
	Fertilizer	Fertilizer use amount/10^4^ t	Fertilizer, pesticide, agricultural film, diesel fuel are the main sources of pollution in the agricultural production process
	Pesticide	Pesticide use amount/10^4^ t	
	Plastic film	Film use amount/10^4^ t	
	Energy	Diesel consumption amount/10^4^ t	
Desired outputs (*y^d^*)	Economic output	Gross agricultural product/100 million CNY	Converted to constant price in 1978 to eliminate the effect of price changes
Non-desired outputs (*y^u^*)	Pollution emission	Agricultural non-point source pollution/10^4^ t	Fertilizer loss, ineffective pesticide utilization, and agricultural film residue

**Table 2:** Variables definition and descriptive statistics.

Variable/Unit		Variable Definition	Mean	Std. Dev.	Min	Max
Explained variable	Agricultural eco-efficiency (AEE)	Measurement based on super-efficient SBM model	0.474	0.334	0.085	2.385
Core explanatory variable	Fertilizer use intensity (FUI)/(kg/hm^2^)	Agricultural fertilizer use/total crop sown area	249.540	139.066	9.170	799.590
Independent variables	Rural Labor Transfer (RLT)/(10^4^ people)	Rural employees—agricultural employees	445.942	497.162	1.400	2226.640
	Disposable income of rural households (DIR)/(CNY)	Disposable income of rural households per capita	4412.624	5548.311	100.930	34,911.000
	Machinery input intensity (MII)/(kW/hm^2^)	Total power of agricultural machinery/total crop sown area	158.396	52.700	43.050	285.850
	Multiple cropping index (MCI)/(%)	Total crop sown area/cropland area	3.795	2.801	0.289	14.156
	Crop planting structure (CPS)/(%)	Grain crop planting area/total crop sown area	70.558	11.952	32.810	97.080
	Fiscal Supporting on Agriculture (FSA)/(CNY/hm^2^)	Fiscal expenditure on agriculture, forestry and water affairs/total crop sown area	9.871	5.298	0.415	67.321

**Table 3:** Unit root test results of variables.

Original Variables	LLC		IPS		ADF-Fisher		Harris-Tzavalis		VIF
	Value	*p*	Value	*p*	Value	*p*	Value	*p*	
lnFUI	−4.435	0.000	−7.933	0.000	−6.033	0.000	0.739	0.000	4.34
lnRLT	−1.753	0.039	−5.043	0.000	−3.924	0.000	0.702	0.000	2.10
lnDIR	−2.213	0.014	−5.563	0.000	−2.428	0.007	0.796	0.031	5.01
lnMII	−1.865	0.023	−0.907	0.182	−3.457	0.000	0.978	0.000	4.12
ln*MCI*	−1.960	0.017	−4.840	0.000	−1.890	0.029	0.692	0.000	1.95
ln*CPS*	−1.417	0.078	−4.314	0.000	−2.923	0.002	0.797	0.033	1.45
ln*FSA*	−4.347	0.000	−9.206	0.000	−7.010	0.000	0.728	0.000	1.11

Note: The different unit root tests all include time trend and subtract the cross-sectional mean.

**Table 4:** Estimation results of the QRM for the impact of FUI on AEE.

Quantile	lnFUI	lnRLT	lnDIR	lnMII	lnMCI	lnCPS	lnFSA	*C*	*R* ^2^
Baseline	−0.338 *** (0.035)	−0.141 *** (0.016)	0.466 *** (0.018)	0.129 *** (0.037)	−0.287 *** (0.053)	−0.304 *** (0.081)	−0.004 (0.022)	−1.897 *** (0.487)	0.606
*θ* = 10%	−0.211 *** (0.026)	−0.124 *** (0.008)	0.387 *** (0.032)	−0.028 (0.021)	−0.131 *** (0.032)	−0.390 *** (0.051)	−0.040 *** (0.018)	−0.118 (0.362)	0.562
*θ* = 20%	−0.154 *** (0.023)	−0.169 *** (0.008)	0.327 *** (0.028)	−0.004 (0.018)	0.012 (0.028)	−0.308 *** (0.045)	−0.044 *** (0.017)	−0.872 *** (0.320)	0.566
*θ* = 30%	−0.171 *** (0.023)	−0.202 *** (0.008)	0.268 *** (0.029)	0.082 *** (0.019)	0.126 *** (0.029)	−0.227 *** (0.047)	−0.068 *** (0.017)	−1.141 *** (0.331)	0.563
*θ* = 40%	−0.163 *** (0.027)	−0.235 *** (0.009)	0.226 *** (0.034)	0.120 *** (0.022)	0.199 *** (0.034)	−0.261 *** (0.054)	−0.080 *** (0.020)	−0.862 *** (0.382)	0.540
*θ* = 50%	−0.157 *** (0.029)	−0.269 *** (0.010)	0.163 *** (0.036)	0.141 *** (0.024)	0.261 *** (0.037)	−0.339 *** (0.058)	−0.136 *** (0.021)	−0.086 (0.411)	0.529
*θ* = 60%	−0.158 *** (0.031)	−0.294 *** (0.010)	0.146 *** (0.038)	0.173 *** (0.025)	0.319 *** (0.039)	−0.358 *** (0.061)	−0.135 ** (0.023)	−0.051 (0.433)	0.524
*θ* = 70%	−0.144 *** (0.035)	−0.316 *** (0.012)	0.148 *** (0.044)	0.168 *** (0.029)	0.341 *** (0.044)	−0.409 *** (0.070)	−0.110 *** (0.026)	0.154 (0.494)	0.514
*θ* = 80%	−0.065 * (0.036)	−0.368 *** (0.012)	0.235 *** (0.046)	0.082 *** (0.030)	0.382 *** (0.046)	−0.517 *** (0.074)	−0.029 (0.027)	1.116 *** (10.97)	0.509
*θ* = 90%	−0.127 *** (0.036)	−0.381 *** (0.012)	0.323 *** (0.046)	0.020 (0.030)	0.298 *** (0.046)	−0.668 *** (0.073)	0.007 (0.026)	1.263 ** (0.518)	0.485

Note: *** *p* < 0.01, ** *p* < 0.05, * *p* < 0.1, and standard errors are in parentheses.

**Table 5:** Estimation results of QRM for the regional grouping.

lnFUI	Economic Sub-Divisions				Sub-Periods		
	ER	CR	WR	NER	1978–1989	1990–2003	2004–2020
Baseline	−0.702 *** (0.060)	−0.445 *** (0.071)	−0.095 * (0.055)	−0.384 *** (0.125)	−0.006 (0.042)	−0.179 ** (0.079)	−0.077 (0.095)
*θ* = 10%	0.029 (0.052)	0.147 ** (0.069)	−0.218 *** (0.045)	−0.185 *** (0.030)	−0.193 *** (0.057)	−0.294 *** (0.054)	0.159 *** (0.039)
*θ* = 20%	0.104 ** (0.049)	0.106 ** (0.049)	−0.118 *** (0.047)	−0.185 *** (0.034)	−0.065 (0.052)	−0.178 *** (0.043)	0.085 ** (0.038)
*θ* = 30%	0.148 *** (0.043)	0.017 (0.048)	−0.101 ** (0.046)	−0.185 *** (0.037)	−0.053 (0.052)	−0.139 *** (0.038)	0.084 ** (0.037)
*θ* = 40%	0.035 (0.040)	−0.013 (0.039)	−0.104 ** (0.046)	−0.224 *** (0.022)	−0.038 (0.050)	−0.126 *** (0.041)	0.126 *** (0.039)
*θ* = 50%	−0.043 (0.041)	−0.044 (0.043)	−0.016 (0.043)	−0.229 *** (0.018)	−0.030 (0.051)	−0.099^**^ (0.044)	0034 (0.037)
*θ* = 60%	−0.091 ** (0.042)	0.032 (0.035)	−0.020 (0.046)	−0.266 *** (0.025)	0.039 (0.050)	−0.129 ** (0.050)	−0.041 (0.039)
*θ* = 70%	−0.136 *** (0.047)	−0.010 (0.048)	−0.061 (0.044)	−0.324 *** (0.046)	0.071 (0.052)	−0.163 *** (0.055)	−0.080 * (0.041)
*θ* = 80%	−0.113 ** (0.053)	0.184 *** (0.054)	−0.131 *** (0.047)	−0.324 *** (0.043)	0.109 ** (0.054)	−0.244 *** (−0.054)	−0.125 *** (0.045)
*θ* = 90%	−0.136 ** (0.062)	0.213 *** (0.058)	−0.171 *** (0.048)	−0.324 *** (0.038)	0.113 ** (0.055)	−0.256 *** (0.060)	−0.187 *** (0.051)

Note: *** *p* < 0.01, ** *p* < 0.05, * *p* < 0.1, and standard errors are in parentheses.

**Table 6:** Estimation results of QRM with the introduction of the spatial lag term.

lnFUI	*Wq*		*Wd*		*Wa*	
	lnFUI	*Wq* × lnFUI	lnFUI	*Wd* × lnFUI	lnFUI	*Wa* × lnFUI
*θ* = 10%	−0.281 *** (0.026)	−0.020 *** (0.003)	−0.166 *** (0.025)	0.691 *** (0.086)	−0.196 *** (0.025)	0.210 *** (0.031)
*θ* = 20%	−0.228 *** (0.023)	−0.016 *** (0.003)	−0.118 ** (0.022)	0.780 *** (0.073)	−0.127 *** (0.021)	0.256 *** (0.026)
*θ* = 30%	−0.223 *** (0.024)	−0.020 *** (0.003)	−0.151 *** (0.024)	0.961 *** (0.078)	−0.166 *** (0.023)	0.329 *** (0.028)
*θ* = 40%	−0.215 *** (0.026)	−0.022 *** (0.003)	−0.138 *** (0.025)	1.170 *** (0.084)	−0.132 *** (0.026)	0.382 *** (0.032)
*θ* = 50%	−0.226 *** (0.026)	−0.024 *** (0.003)	−0.095 *** (0.028)	1.282 *** (0.093)	−0.134 *** (0.028)	0.389 *** (0.035)
*θ* = 60%	−0.201 *** (0.029)	−0.021 *** (0.004)	−0.059 *** (0.028)	1.422 *** (0.095)	−0.116 *** (0.029)	0.426 *** (0.035)
*θ* = 70%	−0.161 *** (0.035)	−0.013 *** (0.004)	0.012 (0.029)	1.508 *** (0.097)	−0.055 *** (0.029)	0.437 *** (0.036)
*θ* = 80%	−0.047 (0.035)	0.009 ** (0.004)	0.022 (0.028)	1.480 *** (0.095)	0.014 (0.030)	0.472 *** (0.037)
*θ* = 90%	−0.134 *** (0.036)	0.007 * (0.004)	0.031 (0.032)	1.561 *** (0.105)	0.057 * (0.033)	0.446 *** (0.041)

Note: *** *p* < 0.01, ** *p* < 0.05, * *p* < 0.1, and standard errors are in parentheses.

## Data Availability

The data presented in this study are available on request from the corresponding author. The data are not publicly available due to data management. The data is also available through public sources.

## Appendix A

Variation of FUI coefficients in economic subdivisions and sub-periods at different quartiles.
